# A flow cytometric assay of murine erythrocyte osmotic fragility

**DOI:** 10.1371/journal.pone.0345170

**Published:** 2026-04-22

**Authors:** Pengwei Zhang, Lishuang Zhang, Shengbao Chen, Yanjuan Chen, Xianmin Zhu, Ruling Shen, Ying Lei

**Affiliations:** 1 Shanghai Institute for Advanced Immunochemical Studies, ShanghaiTech University, Shanghai, China; 2 Department of Orthopaedic Surgery and Institute of Microsurgery on Extremities, Shanghai Sixth People’s Hospital Affiliated to Shanghai Jiao Tong University School of Medicine, National Center for Orthopaedics, Shanghai, China; 3 Shanghai Laboratory Animal Research Center, Shanghai, China; Aga Khan University, PAKISTAN

## Abstract

Flow cytometric test of erythrocyte osmotic fragility (EOF) is a critical diagnostic tool for evaluating membrane stability in hemolytic disorders and storage lesions of human erythrocytes. However, there is a lack of such protocols tailored for mice, one of the most widely used preclinical animal models. In this work, we developed and optimized a flow cytometric assay of murine EOF, which achieved results comparable to the conventional osmotic fragility test. We also validated the protocol by detecting membrane instability of murine erythrocytes induced by Ca² ⁺ -treatment, long-term storage, and phenylhydrazine (PHZ)-induced acute anemia, respectively. Our results showed that Ca² ⁺ -treatment impaired membrane integrity of mouse erythrocytes, resulting in reduced percentage of residual cells compared to the untreated ones (46.5 ± 9.5% vs 79.6 ± 5.3%, *P* < 0.0001). We found that murine erythrocytes can be stored in 1 x PBS containing 2% FBS at 4°C for one week with intact membrane stability (81.6 ± 4.6% of residual cells vs 82.1 ± 6.0% for the fresh samples, p > 0.05). In PHZ induced anemia model, significant hemolysis was observed at day 3 post injection. In conclusion, our flow cytometric assay of murine EOF is a powerful high-throughput assay for mouse models of membrane disorders, and holds substantial scientific and translational value for investigating pathoetiological mechanisms and developing therapeutic interventions.

## 1 Introduction

The mature mammalian erythrocytes have a unique biconcave disc shape, which is maintained by the cytoskeleton network [1,2]. The cell shape and membrane integrity can be impaired by a variety of pathophysiological factors, including genetic defects (e.g., mutations in Ankyrin and Spectrin proteins), metabolic abnormalities (e.g., ATP depletion), oxidative stress (e.g., accumulation of reactive oxygen species), mechanical damage (e.g., vascular shear), and immune attack (e.g., complement-mediated lysis) [3]. It is well known that intracellular calcium plays key roles in maintaining erythrocyte membrane functions such as control of shape, membrane lipid composition and cation permeability [4]. Notably, extracellular Ca^2+^ decreases membrane stability by promoting cross-linking of membrane proteins through activation of transglutaminase [5].

Many methods have been established to detect the mechanical stability of erythrocyte membrane. The conventional erythrocyte osmotic fragility (EOF) test measures the amount of released hemoglobin spectrophotometrically after hemolysis in a series of gradient hypotonic saline solution [6]. Acidified glycerol hemolysis test determines different dissolution rates of erythrocytes in hypotonic glycerol solution [7]. Osmotic gradient ektacytometry measures red cell deformability by laser-diffraction viscometer over an osmotic range [8]. Hyperosmotic cryohemolysis test examines the ability of red blood cells to resist hemolysis in a hyperosmotic environment [9]. The eosin-5’-Maleimide (EMA) binding test was used for the diagnosis of red cell membrane disorders such as hereditary spherocytosis (HS) as the fluorescent dye could label band 3 or Rh-related proteins [10]. If the known biomarkers (e.g., Ankyrin and Spectrin) exist, biochemical methods such as sodium dodecyl sulphate-polyacrylamide gel electrophoresis (SDS-PAGE) and Western blot can reveal their quantitative changes in the complex of cytoskeleton [3]. To simulate microcirculation conditions, deformability of red blood cells (RBCs) can be measured at single cell level inside the microfluidic chips [11]. Among these methods, the EOF test is relatively simple [12] and therefore widely used in the clinical diagnosis of hereditary hemolytic disorders such as hereditary spherocytosis, sickle cell anemia, and thalassemia, and the assessment of erythrocyte storage lesions and drug-induced toxicity [3,13,14]. When coupled with flow cytometry, the so-called flow cytometric EOF test become more quantitative and reliable [15–18] and is gradually replacing the traditional version of method.

Although it has been widely used in clinical diagnosis, the protocols for flow cytometric EOF test are not developed for mice, which are important preclinical models for studies on hematological diseases (e.g., hereditary anemia and malaria infections) because of their clear genetic background, short reproductive cycle, and ease of genetic modification [19]. Instead, the conventional EOF test was still used in mouse models [20–25], which required a large volume of blood and a plethora of procedures.

In this work, we established a flow cytometric assay tailored for examination of EOF in mouse models, the results of which were comparable to the conventional osmotic fragility test. We optimized the conditions of hypotonic treatment, which quantitatively detected membrane instability caused by the Ca^2+^ treatment. We also used this protocol to monitor the membrane integrity of murine erythrocytes during long-term storage and in phenylhydrazine (PHZ) induced acute anemia.

## 2 Materials and methods

### 2.1 Subject of the study

Healthy male C57BL/6 mice (10–12 weeks old) were obtained from Shanghai Southern Model Organisms Center, Inc. The animals were housed at controlled temperature (21 ± 1°C) and humidity (40–60%) with food and water provided *ad libitum* on a 12-hour light/dark cycle. All experiments were performed under the animal protocol approved by Institutional Animal Care and Use Committee at ShanghaiTech University (no. 20250609002).

#### Methods of sacrifice:

After the experiments, euthanasia was performed by exposure to CO2 and then by cervical dislocation in home cages. **Methods of anesthesia**: Blood of 100 µL from each mouse was drawn by retroorbital bleed under light isoflurane anesthesia. **Efforts to alleviate suffering**: The experiments were performed by trained personnel and approved by Institutional Animal Care and Use Committee at ShanghaiTech University. As stated above, anesthesia was performed in the invasive procedures.

### 2.2 Animal experiments and sample collection

For the conventional EOF test, blood samples were collected from total 20 mice (10 controls and 10 mice treated with Ca²⁺). For flow cytometric assay of murine EOF, blood samples were collected from 48 mice (24 controls and 24 mice treated with Ca²⁺). In the control group, fresh erythrocytes analyzed within 2 h of collection. In the Ca² ⁺ treatment group, erythrocytes were incubated with 1.2 mM CaCl_2_ at 37°C for 12 h followed by vortex (500 rpm, 1 min) [5].

For Phenylhydrazine (PHZ) induced acute anemia, total 24 healthy male C57BL/6 mice of 10-wk old were randomly assigned as control group (12 mice) and treatment group (12 mice). In the treatment group, PHZ (MCE) was dissolved in 1x phosphate buffer solution (PBS, Life- I Lab) and administered by the peritoneal injection (pi) at 40 mg/Kg body weight on day 0. Two additional injections were given at 9 am and 4 pm respectively on day 1. The control group was administered with the same amount of 1x PBS. During 0–7 days, we monitored the body weight and did the gross inspection of the mice’s diet, drinking, activity level, mucosal color, and fecal status every day. Peripheral blood was drawn at 0, 3 and 7 days. Red blood cells (RBC), hemoglobin (HGB), HCT, and reticulocyte percentage (RET%) were analyzed in 12 samples (6 controls and 6 PHZ treated mice) by complete blood count (CBC) using ProCyte Dx® Hematology Analyzer (IDEXX Laboratories Inc). The other 12 samples (6 controls and 6 PHZ treated mice) were analyzed by flow cytometric EOF.

### 2.3 Preparation of erythrocyte suspension

Mouse whole blood of 50 μL was taken with addition of 1.5 mg/mL EDTA to prevent coagulation. The blood was washed twice with 1x PBS containing 2% fetal bovine serum (FBS, Gibico) at 300xg, 4°C for 5 min. The erythrocyte pellets were resuspended with 100 μL of 1x PBS containing 2% FBS. Erythrocytes of 50 μL was added into 450 μL of PBS containing 2% FBS.

### 2.4 The conventional EOF test

Nine test tubes containing gradient NaCl concentrations (prepared with 9 g/L NaCl (Servicebio) and sterile water (ddH_2_O); the first Table in S1 Appendix) were mixed with 50 μL erythrocytes respectively. After 30 min of incubation at 20°C (with one gentle mixing at 15 min), samples were centrifuged (2500 × g, 5 min). Supernatant absorbance (200 μL) was measured at 540 nm using BioTek CYTATION3 (Agilent). The value of 1 g/L NaCl was used as 100% for complete hemolysis [5,15]. The corresponding percentage of hemolysis was calculated from the Absorbance (A) value of each tube. Calculation formula:


Percentage of hemolysis (%) = (Measurement tube A-value/completely hemolyzed tube A-value) × 100%

In order to make a comparison with the flow-through test results, the formula was changed to:


Residual red cells (%) = 100% - Percentage of hemolysis (%)

### 2.5 Instrument calibration and sample preparation

All assays were performed on a FACSAria^TM^Ш flow cytometer (BD Biosciences), which was calibrated daily using standardized CST Beads [16]. Sample preparation was as follows: 1.1 mL of 1xPBS containing 2% FBS was added to the flow tube. Then 5 μL of erythrocyte suspension was accurately pipetted and added vertically to the bottom of the tube (avoiding adding along the wall of the tube). The erythrocyte suspension was made by gently mixing and would be used for flow cytometric analysis.

### 2.6 Flow detection parameter settings

EOF was assessed using linear amplification mode for FSC/SSC measurements. A time-resolved FSC analysis was performed over 170 seconds (divided into 8 gates, i.e., R1-R8, about 11 seconds/gate) [16]. After initiating acquisition, the test was paused following R1 completion. After 450 μL of water until all 8 gates were completed.

The degree of osmotic hemolysis was quantitatively analyzed by the change of cell counts in each interval (R1-R8) in the TIME-FSC scatter plot. The percentage of residual red cells was used as a key index for EOF and calculated as follows:


Residual red cells (%) = [(number of cells in gate R7 + number of cells in gate R8)/2]/ [number of cells in gate R1 × 1.1/(1.1 + 0.45)] × 100%

The R1 gate provided the baseline erythrocyte count, while subsequent gates (R2-R8) monitored osmotic hemolysis dynamics. Residual erythrocyte percentage was calculated using R7 and R8 gates, applying a 1.1/1.55 correction factor to account for volume dilution (from 1.1 mL to 1.55 mL) following 0.45mL of water spike-in.

### 2.7 Statistical methods

All flow cytometric data were analyzed using FlowJo software. After D’ Agostino and Pearson’s omnibus normality test, the data were statistically analyzed using two-way ANOVA or paired t-test with 95% confidence interval. All data were referred to as Mean±SD. Differences were considered statistically significant when *P* < 0.05. All graphics, calculations, and statictical analyses were performed using Graphpad Prim 10.4.0 software.

## 3 Results

### 3.1 Effect of Ca^2+^ on membrane stability detected by the conventional EOF test

**Fig 1:**
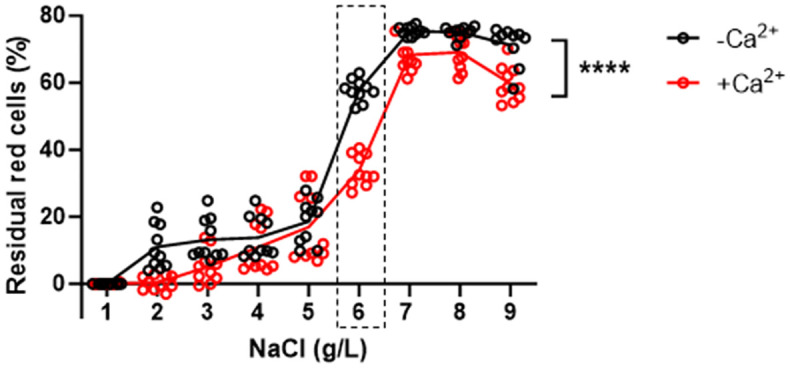
Validation of membrane instability induced by Ca^2^^+^ treatment using the conventional EOF test (n = 10 for each group). The percentage of residual red cells were calculated by the standard curve (The first Figure in S1 Appendix) at different NaCl concentration. The dashed-line rectangle highlighted that there was the largest Mean difference (23.9%) of residual cell percentage between treated and untreated samples at 0.6% NaCl (*P <* 0.0001).

As flow cytometric EOF was seldom tested in mice, we decided to initially examine it by the conventional EOF test. We chose Ca^2+^ as the perturbation factor for membrane stability. After establishing a standard curve correlating erythrocyte number with hemoglobin absorbance at 540nm (The first Figure in S1 Appendix), we measured EOF of total 10 mice before and after Ca^2+^treatment (Fig 1). For human samples, the initial hemolysis usually occurs at 0.42–0.46% NaCl (4.2–4.6 g/L) and the complete lysis at 0.28–0.34% NaCl (2.8–3.4 g/L) [15]. Interestingly, our results showed that healthy murine erythrocytes (untreated) began hemolysis at 0.60% NaCl (Fig 1, the raw data and statistics in S2 Table) and reached the plateau of complete lysis at 0.50% NaCl (Fig 1, the raw data and statistics in S2 Table), indicating that their tolerance to hypotonic solutions were lower than human beings. This may be likely due to murine erythrocytes’ smaller size and higher surface area-to-volume ratio, which diminish their resistance to osmotic stress [26,27]. After Ca^2+^ treatment, the percentage of residual cells was significantly reduced compared to the untreated samples at different concentrations of NaCl (P of column factor < 0.0001, Fig 1A), indicating that membrane stability was impaired by Ca^2+^ treatment. At 0.6% NaCl, the percentage of residual cells in Ca^2+^ treated samples was significantly lower than the untreated samples (33.9 ± 4.7% *vs* 57.8 ± 3.3%, *P* < 0.0001, the raw data and statistics in S2 Table). Using Annexin V binding assay (The second Figure in S1 Appendix), we found that calcium induced hemolysis was not associated with apoptosis-like features (eryptosis).

Taken together, mouse erythrocytes have low tolerance to hypotonic solutions and their membrane stability is impaired by Ca^2+^ treatment, which can be quantified by the conventional EOF test.

### 3.2 Acquisition parameters for flow cytometric EOF test

**Fig 2:**
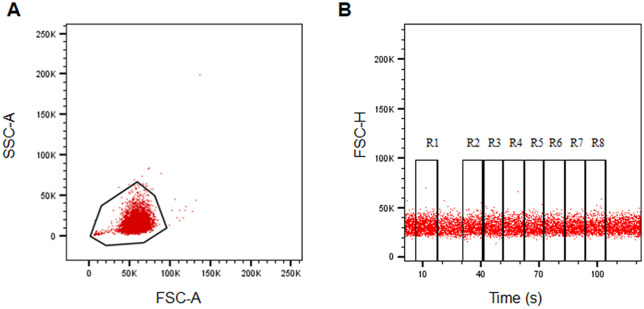
Gating method for flow cytometric assay of mouse EOF (A) A representative figure showing mouse erythrocytes were collected using a linear amplification mode gating; (B) A representative figure showing time-continuous gating with FSC-H as the vertical axis and time as the horizontal axis.

To develop the protocol of flow cytometric EOF test for mice, we chose BD FACSAria^TM^Ш (Materials and Methods) because its sample acquisition is achieved by air pressure which allows continuous acquisition during the experiment. To enable precise discrimination of erythrocyte subsets, we used linear amplification gating to prevent signal compression of small cell populations in logarithmic scale (Fig 2A). The gating of 8 regions (R1 to R8) before and after water spike-in was made in a time-FSC acquisition plot (Fig 2B). The percentage of residual cells after hemolysis was calculated as stated in Materials and Methods.

### 3.3 Optimization of flow cytometric EOF test for mice

**Fig 3:**
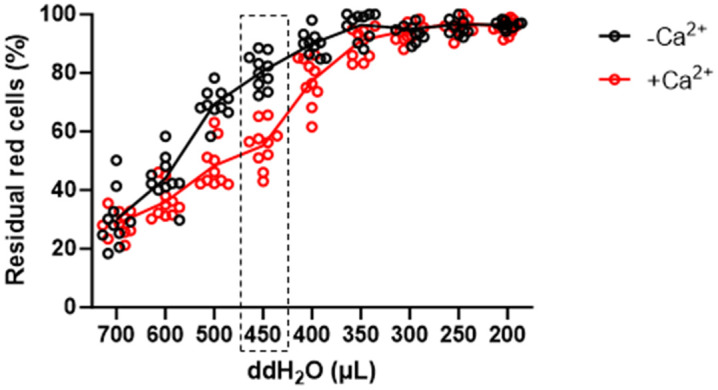
Optimization of flow cytometric EOF test for mice. Comparison of residual cells between Ca² ⁺ -untreated and -treated murine erythrocytes (n = 10 for each group) after adding different volumes of water (ddH_2_O). The dashed-line rectangle highlighted that there was the largest Mean difference (25.5%) of residual cell percentage between treated and untreated samples at addition of 450 μL water (ddH₂O) (*P <* 0.0001).

In human samples, water spike-in of 0.9 mL into the 1.1mL of red cells can induce hemolysis [16,17]. As stated above, murine erythrocytes have low tolerance to hypotonic solutions (Fig 1). To determine the optimized amount of water spike-in for mice which could distinguish membrane disorders (i.e., Ca² ⁺ treatment in this case) from the healthy controls, we added different amount of water spike-in ranging from 0.2 to 0.7 mL in the flow cytometric EOF test using the same samples as the conventional EOF test. As expected, the curve of residual red cells in flow cytometric assay of EOF had similar pattern compared to that of the conventional EOF test. The percentage of residual cells after hemolysis was negatively correlated with the amount of water spike-in (Fig 3). This was consistent with the results by the conventional EOF test (Fig 1), We observed a significant difference in the percentage of residual cells between Ca² ⁺ -treated and non-Ca² ⁺ -treated mouse erythrocytes as analyzed by two-way ANOVA when the amount of water added ranged from 350 to 600 μL (Fig 3, the raw data and statistics in S2 Table). It should be noted that the residual cells varied considerably with the volume of water added and the largest Mean difference between treated and untreated samples was observed at addition of 450 μL water (55.2 ± 7.4% with Ca² ⁺ treatment *vs* 80.7 ± 5.7% in untreated samples, *P* < 0.0001; Fig 3, representative time/FSC plots in the third Figure in S1 Appendix). Taken together, we optimized the flow cytometric EOF test particularly for mice and used water spike-in of 450 μL in the experiments thereafter.

### 3.4 Detection of membrane instability of murine erythrocytes by flow cytometric EOF test

**Fig 4:**
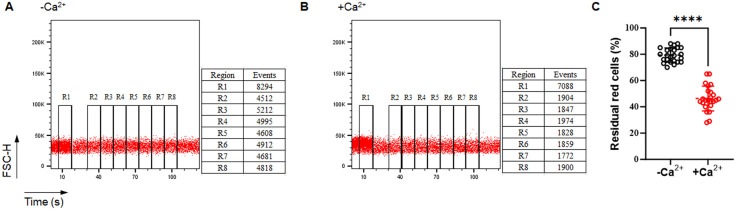
Detection of membrane instability of murine erythrocytes induced by Ca² ⁺ treatment using flow cytometric EOF test for mice. Shown were the representative figures of flow cytometric detection of residual erythrocytes without Ca^² ⁺^ treatment (A), and with Ca^² ⁺^  treatment (B) respectively; (C) The statistical comparison of the percentage of residual cells in Ca^² ⁺^  -untreated and treated murine erythrocytes. ****P < 0.0001, n = 24 for each group.

We next decided to use the flow cytometric assay of murine EOF to examine membrane instability of murine erythrocytes caused by Ca² ⁺ -treatment. We performed flow cytometric EOF assay using peripheral blood in a larger sample size from 24 C57BL/6 mice. The data were collected for the fresh samples (the untreated group) and the samples threated with CaCl_2_ at 37℃ for 12h. The data of both groups were normally distributed (D’ Agostino and Pearson’s omnibus normality test, *P* > 0.05). Consistently, our results showed that Ca^2+^ treatment significantly impaired membrane stability of mouse erythrocytes (46.5 ± 9.5% with Ca^2+^ treatment *vs* 79.6 ± 5.3% in untreated samples, *P* < 0.0001; Fig 4, the raw data and statistics in S2 Table). We calculated the effect size and found huge difference between the two groups (Cohen’s d = 4.30). Taken together, membrane instability of murine erythrocytes (e.g., Ca^2+^ treatment) can be detected by flow cytometric EOF test for mice.

### 3.5 Detection of storage lesion of murine erythrocytes by flow cytometric EOF test for mice

Membrane stability will be gradually decreased as erythrocytes is stored for a long period of time. The so-called storage lesion is usually determined by EOF tests. Therefore, we decided to test if our flow cytometric assay of murine EOF could be used to detect storage lesion for mouse samples. We used 1x PBS containing 2% FBS to store mouse erythrocytes at 4°C for 28 days and performed flow cytometric assay of EOF at different time points, i.e.,: 2 h (fresh samples), 24 h, 168 h (7 days), 336 h (14 days), 504 h (21 days), and 672 h (28 days).

**Fig 5:**
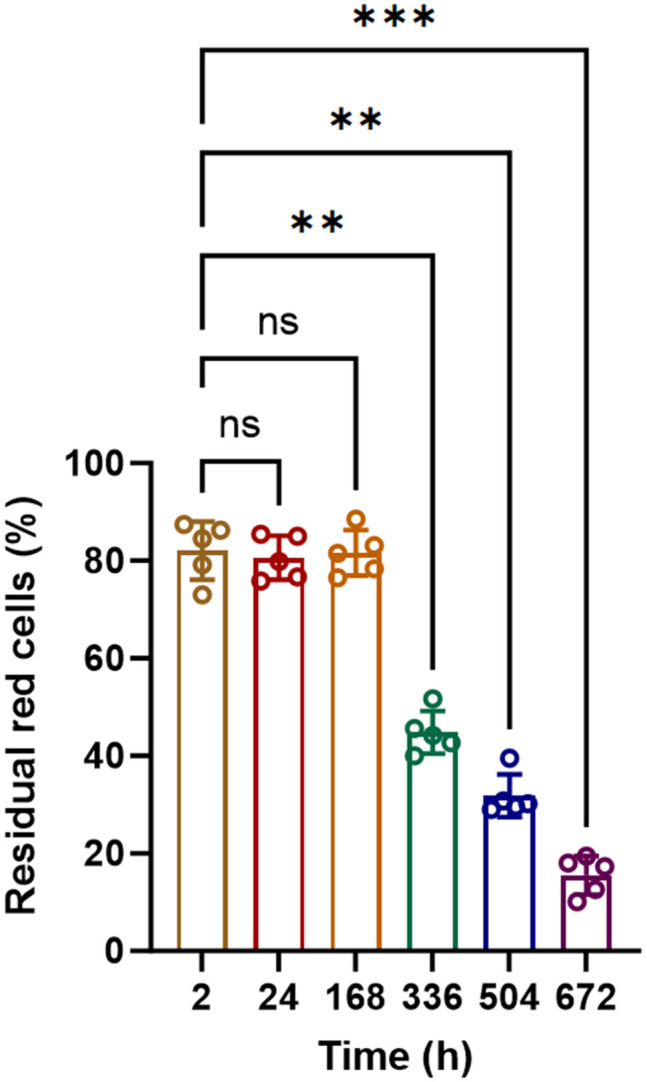
Detection of storage lesion of murine erythrocytes by flow cytometric assay of murine EOF. Murine erythrocytes (n = 5) were stored at 4°C for total 28 days in 1X PBS containing 2% FBS. The percentage of residual red cells was determined by flow cytometric assay of EOF at different time points, i.e.,: 2 h, 24 h, 168 h (7 days), 336 h (14 days), 504 h (21 days), and 672 h (28 days). **P < 0.01; ***P < 0.001.

During the initial 168 hours (7 days) of storage, mouse erythrocytes maintained excellent osmotic stability with about 80% of residual cells, which showed no significant difference from the fresh samples (*P* > 0.05; Fig 5, representative time/FSC plots in the fourth Figure A-C in S1 Appendix, the raw data and statistics in S2 Table). However, prolonged storage for 336 hours (14 days) resulted in a significant increase in osmotic fragility (44.9% ± 4.4%, *P* < 0.0001, Fig 5, representative time/FSC plot in the fourth Figure D in S1 Appendix, the raw data and statistics in S2 Table). At 504 hours (21 days), the percentage of residual cells decreased to 31.8% ± 4.3% (*P* < 0.0001 compared to 168 hours, Fig 5, the fourth Figure E in S1 Appendix, the raw data and statistics in S2 Table). At the final time point (672 hours, 28 days), the percentage of residual red cells was significantly reduced to 15.5% ± 4.0% (*P* < 0.0001 compared to 168 hours, Fig 5, representative time/FSC plots in the fourth Figure F in S1 Appendix, the raw data and statistics in S2 Table).

Taken together, our flow cytometric EOF test could be used as a reliable and sensitive method to detect storage lesion of mouse erythrocytes, which showed that murine erythrocytes could be well preserved in 2% FBS/PBS at 4°C with optimal osmotic stability for a week.

### 3.6 Detection of hemolysis in PHZ-induced acute anemia mouse model by flow cytometric EOF test

**Fig 6:**
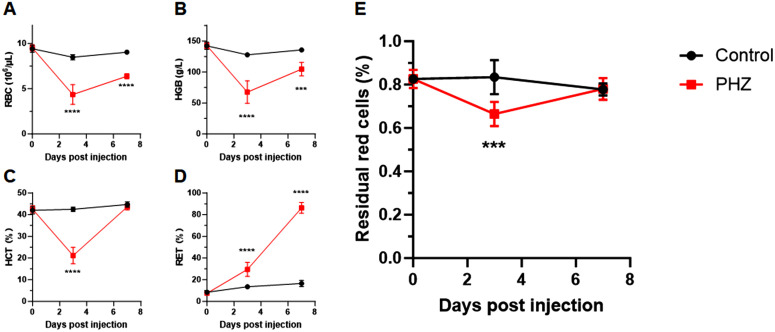
Detection of hemolysis in PHZ-induced acute anemia in mice by flow cytometric assay of murine EOF. Murine RBC **(A)**, HGB **(B)**, HCT (C) and RET (D) were analyzed at day 0, day 3 and day 7 post injection of PHZ (n = 6). The percentage of residual red cells (E) was determined by flow cytometric EOF assay at day 0, day 3 and day 7 post injection. ***P < 0.001; ****P < 0.0001. RBC, red blood cells; HGB, hemoglobulin; HCT hematocrit; RET, reticulocytes.

As a strong oxidant, PHZ are usually used to generate animal models of acute hemolytic anemia. We decided to validate our flow cytometric EOF protocol in detecting hemolysis in this *in-vivo* model. After pi administration of PHZ, we monitor RBC number, HGB, HCT, and RET% until 7 days post injection. Consistent with a previous report [28], RBC number (Fig 6A) and HGB level (Fig 6B) significantly decreased with the maximal drop at day 3 post PHZ injection, which gradually increased but did not reach the same level as the controls at day 7 post injection. In contrast, HCT significantly decreased at day 3 post PHZ injection, but reached the same level as the controls at day 7 post injection (Fig 6C). This process was accompanied by a rapid reticulocytosis which had an exponential increase until day 7 post injection (Fig 6D). We speculated that, in the acute anemia induced by PHZ, the membrane of RBC was damaged dramatically at day 3 post injection and gradually recovered at day 7. We performed the flow cytometric EOF assay to evaluate the membrane damage by measuring hemolysis during the process of PHZ induced acute anemia. Consistently, the percentage of residual red cells had a significant decrease at day 3 post injection and then returned to the same level as the controls (Fig 6E).

Taken together, the flow cytometric assay of murine EOF offers a great tool to quantitate the membrane functions of RBC in the mouse models of membrane disorders.

## 4 Discussion

Abnormal EOF represents a hallmark pathological manifestation in various hematologic disorders, including hereditary membrane-deficient disorders such as hereditary spherocytosis (HS), sickle cell anemia, and elliptocytosis, as well as acquired erythrocyte storage lesion [25]. Flow cytometry (FCM) has emerged as a powerful and quantitative tool for diagnosis of erythrocyte membrane disorders. The high sensitivity of FCM allows it to not only detect the diseases caused by deficient membrane proteins (e.g., ankyrin, band 3 protein and spectrin), but also distinguish HS from other hereditary hemolytic anemias, e.g., hereditary pyropoikilocytosis (HPP) [29,30]. In addition, FCM-based approaches showed great diagnostic potential for erythrocyte membrane abnormalities such as hereditary elliptocytosis (HE) and hereditary stomatocytosis (HSt) [31–34].

Although FCM coupled EOF tests were widely used for human erythrocytes, an optimized protocol had not been developed for mouse models, one of the most common preclinical animal models, which was likely due to limited knowledge of murine hemolysis characteristics and laborious optimization. Here, this work generated and optimized a specialized flow cytometric assay of EOF for mouse samples. As murine erythrocytes have low tolerance to hypotonic solutions, we added 450 μL of water into 1.1 mL of murine erythrocytes instead of 900 μL for human erythrocytes [17]. We also showed its applications in detecting membrane disorders (e.g., Ca^2+^ treatment) and storage lesion. The limitations of this study include relatively small sample size and lack of experiments on genetically modified disease models (e.g., spherocytosis), which will be further investigated in our future experiments.

Unlike the conventional EOF test which indirectly determines the number of erythrocytes by hemoglobin absorbance during hemolysis, the flow cytometric EOF test directly counts residual erythrocytes in hypotonic conditions, which increases accuracy and eliminates time-consuming steps such as serial dilution and making standard curves. Our results showed that the flow cytometric assay of murine EOF obtained comparable results of the conventional osmotic fragility test when studying membrane instability induced by Ca^2+^ (Figs 1 and 3). Besides, the flow cytometric assay of EOF is suitable for high-throughput applications because it demands extremely low amount of samples (5 μL of whole blood for each sample, and no more than 5 minutes for each sample).

The flow cytometric assay of murine EOF provides a great tool to detect both *in-vitro* and *in-vivo* membrane disability. PHZ has long been used to generate animal models of acute hemolytic anemia. As a strong oxidant, its metabolites such as phenyldiazene can oxidize hemoglobulin, resulting in the formation of Heinz bodies which is associated with hemolysis. [35] In addition to the damage on hemoglobulin, PHZ induces a decrease of membrane skeleton proteins such as Spectrin, which impairs RBC deformability. [36] Therefore, the anemia animal model induced by PHZ also provided a partial model to study beta-thalassemia. [37] A previous study showed that the decrease of hemoglobulin level reached maximal at day 3 post injection of PHZ, while PHZ induced reticulocytosis was significantly increase and reached plateau at day 7. We observed similar phenomena (Fig 6A-6D). Interestingly, HCT of PHZ treated mice was significantly lower than that of the controls at day 3, but became no difference at day 7 (Fig 6C). Our results suggested that the adverse effects of PHZ were mostly accumulated around day 3 post treatment, and then gradually disappeared after day 7. Consistently, in the flow cytometric EOF assay, we found that the percentage of residual red cells in PHZ treated mice was significantly lower than that in the controls at day 3 post injection, while there was no difference at day 7 (Fig 6E). Nevertheless, our results proved that flow cytometric assay of murine EOF was capable of detecting RBC abnormalities related to membrane disability.

As known, many human membrane disorders, including SCD [38], HS, and HE [39], are modeled in mice. Therefore, our flow cytometric EOF test holds substantial scientific and translational value for investigating pathoetiological mechanisms and developing therapeutic interventions.

## Supporting information

S1 Appendix Supplementary Materials. (S1 Table, S1-4 Figures).
(DOCX)

S2 Table The raw data and statistics of Figure 1, Figure 3, Figure 4, Figure 5, and Figure 6, respectively. (XLSX)
