# Supplementary material for: A flow cytometric assay of murine erythrocyte osmotic fragility
Source: PLoS One. 2026 Apr 22;21(4):e0345170. doi: 10.1371/journal.pone.0345170 (PMC13102304; doi:10.1371/journal.pone.0345170)
Supplement: S1 Appendix — (S1 Table, S1-4 Figures). (DOCX) [file pone.0345170.s001.docx]

**S1 Appendix.** SUPPLEMENTARY MATERIALS

**A flow cytometric assay of murine erythrocyte osmotic fragility**

**S1 Table.** The gradient concentrations of NaCl solution used in the conventional Sanford method.

| NaCl (g/L) | 9 | 8 | 7 | 6 | 5 | 4 | 3 | 2 | 1 |
| --- | --- | --- | --- | --- | --- | --- | --- | --- | --- |
| 9g/L NaCl (μL) | 500 | 444 | 389 | 333 | 278 | 222 | 167 | 111 | 56 |
| ddH2O (μL) | 0 | 56 | 111 | 167 | 222 | 278 | 333 | 389 | 444 |

**S2 Table.** The raw data and statistics of Figure 1, Figure 3, Figure 4, Figure 5 and Figure 6 respectively. (Supplementary Table S2.xlsx).


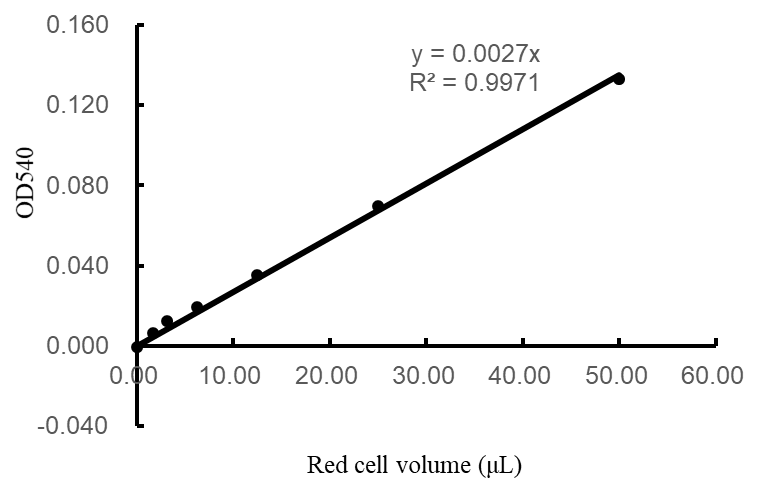


**S1 Figure.** The standard curve showing correlation between hemoglobin absorbance at 540 nm and complete lysis of different volumes of mouse erythrocytes.


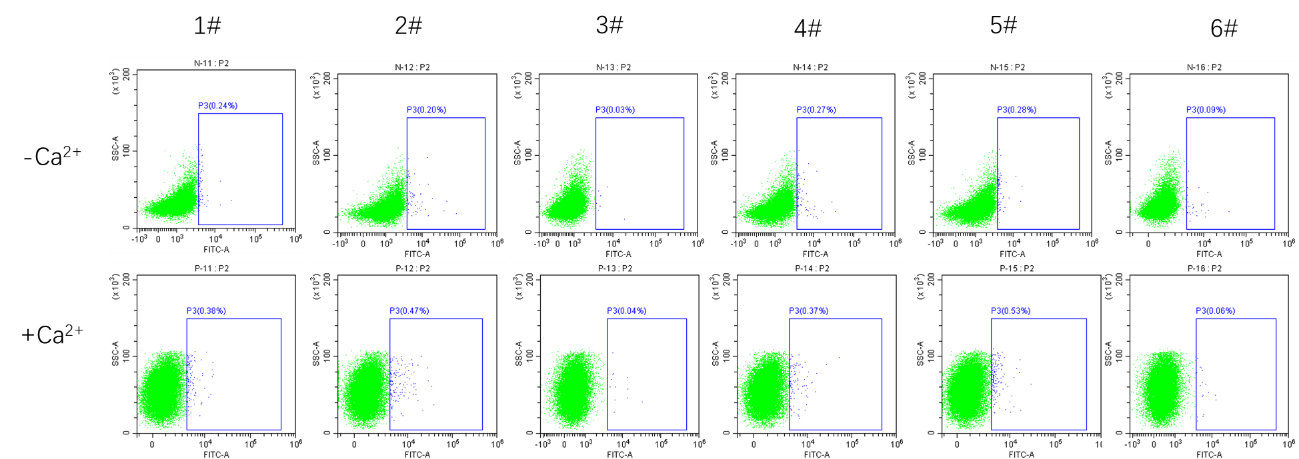


**S2 Figure.** Ca²⁺-mediated eryptosis was not detected by Annexin V binding assay.


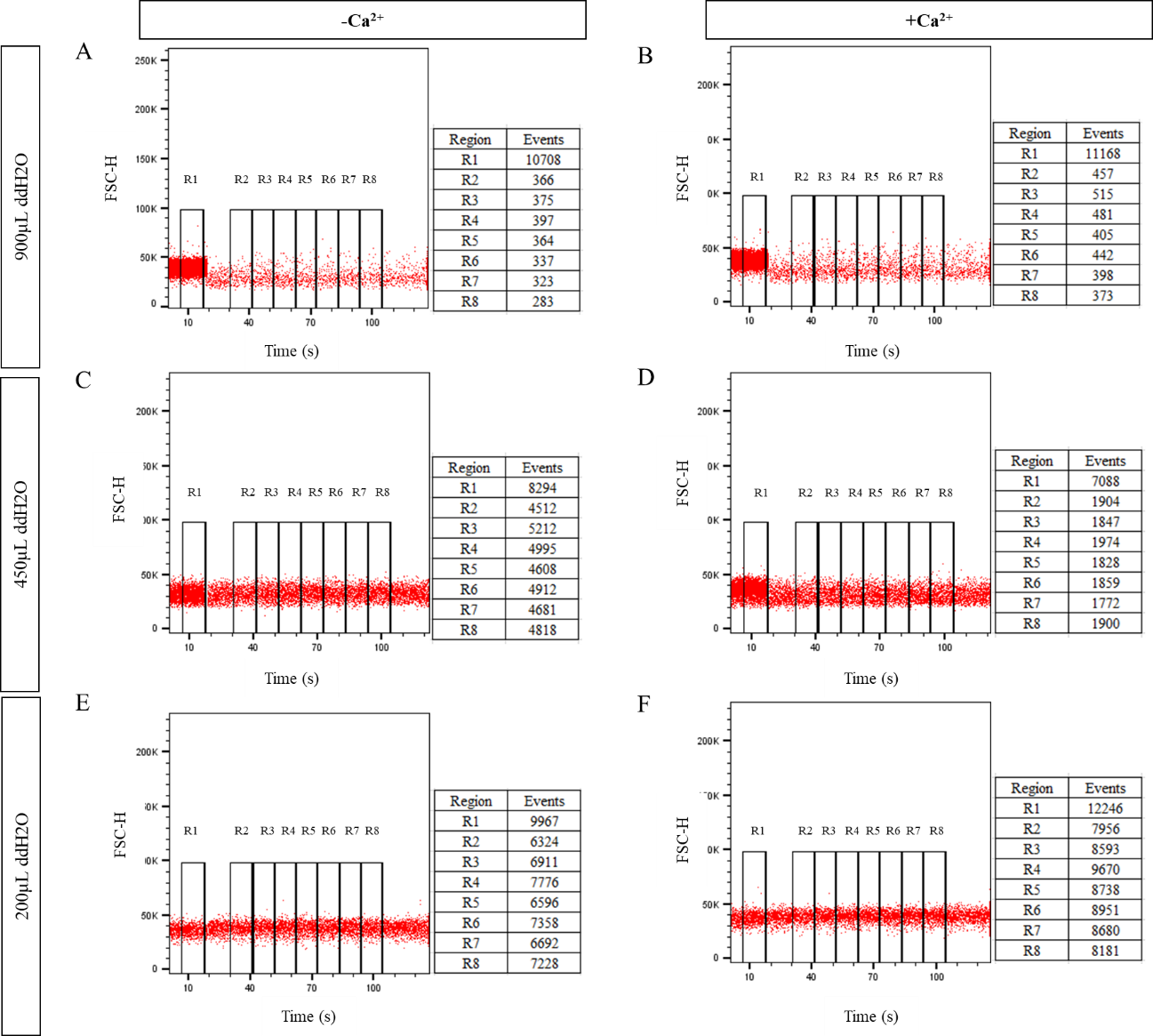


**S3 Figure.** Optimization of the experimental conditions for detection of EOF by flow cytometry. (A) Percentage of residual cells in Ca^2+^-untreated erythrocytes after 900 μL of water spike-in; (B) Percentage of residual cells in Ca^2+^-treated erythrocytes after 900 μL of water spike-in; (C) Percentage of residual cells in Ca^2+^-untreated erythrocytes after 450 μL of water spike-in; (D) Percentage of residual cells in Ca^2+^-treated erythrocytes after 450 μL of water spike-in; (E) Percentage of residual cells in Ca^2+^-untreated erythrocytes after 200 μL of water spike-in; (F) Percentage of residual cells in Ca^2+^-treated erythrocytes after 200 μL of water spike-in.


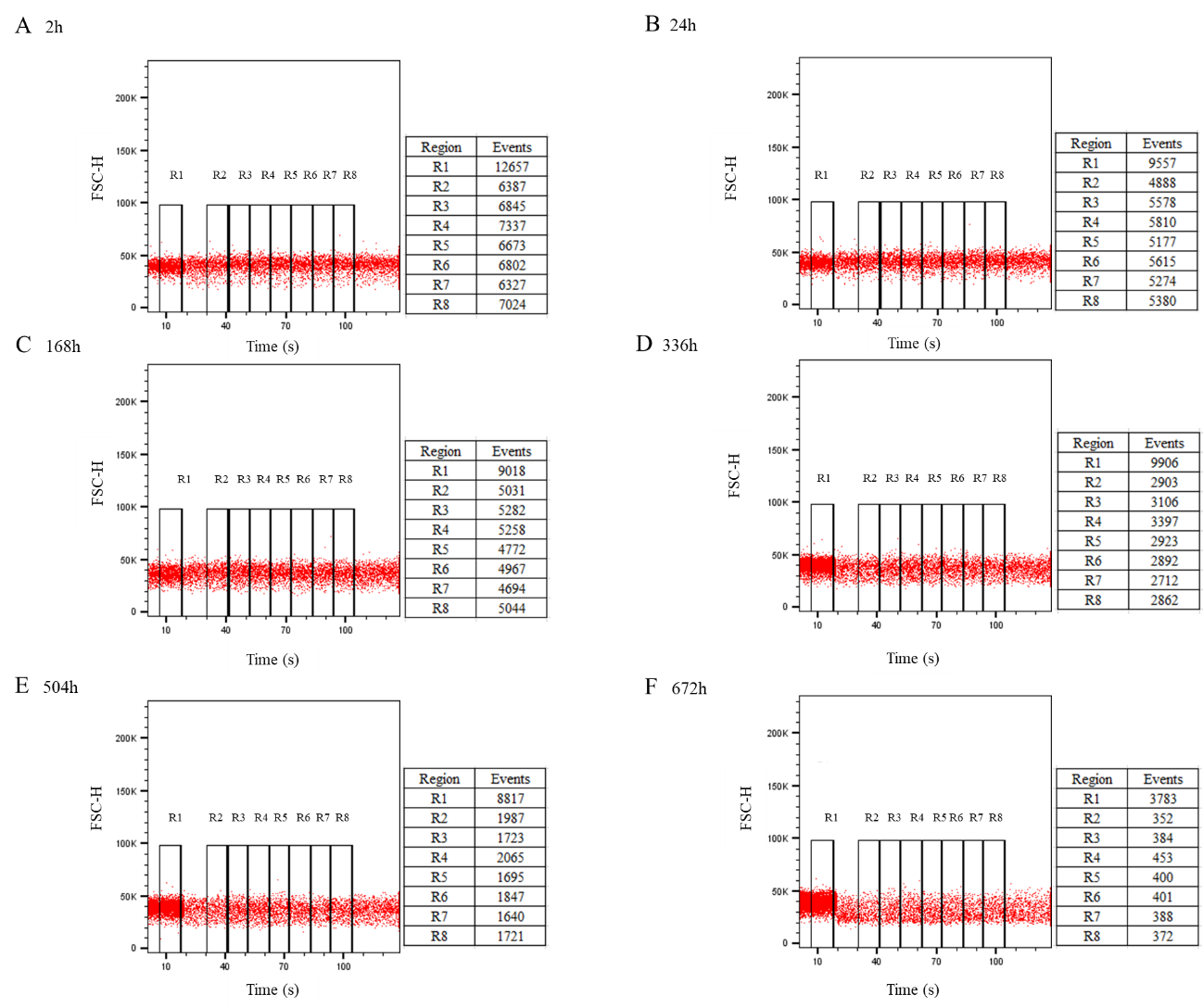


**S4 Figure.** The representative figures of flow cytometric detection of storage lesion of murine erythrocytes at 4°C for total 28 days in 1X PBS containing 2% FBS. The percentage of residual red cells was determined by flow cytometric assay of EOF at 2 h (A), 24 h (B), 168 h (C), 336 h (D), 504 h (E), and 672 h (F).
